# Linking toxicity and predation in a venomous arthropod: the case of *Tityus fuhrmanni* (Scorpiones: Buthidae), a generalist predator scorpion

**DOI:** 10.1590/1678-9199-JVATITD-2021-0036

**Published:** 2022-01-07

**Authors:** Alejandra Arroyave-Muñoz, Arie van der Meijden, Sebastián Estrada-Gómez, Luis Fernando García

**Affiliations:** 1 Toxinology Research Group - Serpentarium, University of Antioquia (UdeA), Medellín, Antioquia, Colombia.; 2 Basic Sciences Development Program (PEDEClBA), University of the Republic, Iguá, Montevideo, Uruguay.; 3 Research Center in Biodiversity and Genetic Resources (CIBIO), University of Porto, Vila do Conde, Portugal.; 4 Eastern Regional University Center (CURE), University of the Republic, Treinta y Tres, Uruguay.; 5 Center for Research in Natural Resources and Sustainability (CIRENYS), Bernardo O’Higgins University, Santiago, Chile.

**Keywords:** Trophic ecology, 50% Lethal dose, Predatory behavior, Scorpions, Tityus

## Abstract

**Background:**

Scorpions are arachnids that have a generalist diet, which use venom to subdue their prey. The study of their trophic ecology and capture behavior is still limited compared to other organisms, and aspects such as trophic specialization in this group have been little explored.

**Methods:**

In order to determine the relationship between feeding behavior and venom toxicity in the scorpion species *Tityus fuhrmanni*, 33 specimens were offered prey with different morphologies and defense mechanisms: spiders, cockroaches and crickets. In each of the experiments we recorded the following aspects: acceptance rate, immobilization time and the number of capture attempts. The median lethal dose of *T. fuhrmanni* venom against the three different types of prey was also evaluated.

**Results:**

We found that this species does not have a marked difference in acceptance for any of the evaluated prey, but the number of capture attempts of spiders is higher when compared to the other types of prey. The immobilization time is shorter in spiders compared to other prey and the LD_50_ was higher for cockroaches.

**Conclusions:**

These results indicate that *T. fuhrmanni* is a scorpion with a generalist diet, has a venom with a different potency among prey and is capable of discriminating between prey types and employing distinct strategies to subdue them.

## Background

Predators often possess adaptations that enhance prey capture. These can include morphological, behavioral, and physiological adaptations. One of the most interesting adaptations for prey capture is the production of different types of secreted proteins that facilitate prey handling by leading to paralysis, death and sometimes pre-digestion of the prey [1,2,3,4]. The role of venom in prey capture has been studied most extensively in vertebrates such as snakes, where venom composition is related to diet breadth and has a high specificity against certain prey taxa [5,6,7]. Despite there being many more venomous groups of invertebrates than vertebrates, studies linking the predatory behavior and toxicity are limited among invertebrates.

In arthropods, most of the studies on the use of venom in the capture of prey have been carried out in spiders. For example, spiders were shown to regulate their venom use according to the type of prey and its resistance [8,9,10]. Venoms can also be highly specific, such as in spiders of the genus *Zodarion* that are specialized in ants [11]. Venoms can also have a broad spectrum in generalist predators, being effective against a wide variety of prey taxa, including vertebrates and invertebrates [2,12]. For example, venomous generalist predators like centipedes possess toxins able to overcome small vertebrates such as mice [13], but are also effective against other arthropods such as insects [14].

To understand the ecological role of venom and the selective pressures that have led to its target specificity, knowledge of the trophic niche and feeding ecology in venomous animals is particularly important. Understanding the relationship between the ecological function and target specificity of venom may also be medically relevant, as some studies hypothesize that toxicity towards humans in some arthropod predators might be a consequence of toxins that target vertebrates as prey [15]. Despite diet having a strong influence in some predatory venomous arthropods, the degree to which venoms are specialized to the preferred prey is still unknown in several groups.

At least three components can be directly associated with predation and defensive behaviors in scorpions: (1) morphology of the chelae and structure of the chelae fingers granulations; (2) morphology of the metasoma and in particular of the telson; (3) evolution of tegumentary glands in the telson toward different types of venom glands [16], the latter being one of the most studied aspects [17]. Despite venom playing a key role in prey capture, most studies have looked at the effect of venom from a defensive perspective in scorpions [18, 19, 20, 21]. Some studies show that the venomous stinger is most used when capturing larger prey, suggesting venom could be optimized to handle difficult or potentially dangerous prey [22, 23, 24, 25]. 

Trophic ecology in scorpions has been poorly studied, with most studies based on relatively sparse field observations. Several of these studies show that scorpions are dietary generalists [26, 27, 28, 29]. Only few studies have explored adaptations for prey capture. For example, Simone et al. [23] showed that females of the scorpion *Bothriurus bonariensis* display distinct prey capture strategies depending on prey type, and consume arthropods with contrasting morphologies and different defensive capabilities, while some other authors have shown intersexual variations on prey capture efficiency in some scorpion species [30]. Regarding trophic specialization, Toscano-Gadea and Costa [31], suggested that the scorpion *Tityus uruguayensis* might be a spider-specialist, given it has specific adaptations for capturing these prey. 

The scorpion *Tityus fuhrmanni* (Krapelin 1914) is an endemic species of the Antioquia department in Colombia [32]. It causes a relatively high number of accidents in the city of Medellin [33]. Although some studies have covered some aspects of the biology of this species, such as life history, distribution, epidemiology, habitat and post-embryonic development [33, 34, 35], the feeding behavior of *T. fuhrmanni* has been poorly explored. 

Since prey capture is strongly linked to venom composition in venomous predators, and given that scorpions depend on their venom delivery and composition to capture different prey, we expect that *T. fuhrmanni* may also possess specialized venoms to deal with certain prey types. If such specialization is present, we would expect the *T. fuhrmanni* to prefer certain prey, and handle those more successfully than others. However, if *T. fuhrmanni* have generalist trophic habits, as described by most previous studies in other scorpions, we expect a similar acceptance, predatory efficiency, and venom toxicity against all the evaluated prey. Therefore, the aim of this study is to compare prey acceptance, predatory efficiency, and venom toxicity in the scorpion *T. fuhrmanni* against different prey types: cockroaches, crickets and spiders. These prey possess different defensive strategies, such as fast movements in spiders and crickets, and venomous fangs in spiders, whereas cockroaches besides quick sprints possess tough cuticles too [38, 39, 40]. Overcoming such defensive strategies effectively may require a certain level of specialization by the predator.

## Methods

### Specimen collection and housing

We collected 33 individuals of *Tityus fuhrmanni* (9 males, 17 females and 7 juveniles) in Antioquia, Colombia, in a locality next to the village "El Salado" (6° 21' 18.4" N 75° 28' 50.4" W), and in the Medellín region on “El Volador” hill (6° 15' 47.4" N 75° 34' 55.3" W). We selected these individuals based on their local abundance.

The Scorpions were deposited in the Serpetarium of the University of Antioquia, as part of the living collection (COLVIOFAR-149). After collection, specimens were kept individually in plastic terrariums (21 cm x 15 cm x 6 cm height) with moist soil as a substrate and tree bark as a refuge. We also emulated the humidity (70 ± 10%), temperature (22 ± 5°C) and photoperiod (12-hour light/12-hour dark) of the sampling locality. 

To select the prey types, we chose arthropods previously reported in the diet of other scorpion species [36], and that were sympatric with the local populations of *T. fuhrmanni*, namely, spiders (*Ctenus* sp., Araneae: Ctenidae), cockroaches (*Periplaneta americana,* Linnaeus, 1758, Blattodea: Blattidae) and crickets (*Acheta domesticus* Linnaeus, 1758, Orthoptera: Gryllidae). The latter were not the same species as encountered in the field, but are of the same genus, and similar in size and overall shape. Prey were selected based on their different morphology and defensive strategies, where crickets have fast movements, kicking and autotomy as their main defense mechanisms, cockroaches present a tough cuticle, while spiders can retaliate against the attack of potential predators by using their venomous chelicerae [37, 38, 39]. To avoid potential bias because of prey size, all prey were selected to be about three times larger than the size of the scorpion’s prosoma (Table 1). Morphological measurements were made from digital photographs using the software imageJ version 1.8.0 [40].

**Table 1.** Mean lengths of *Tityus fuhrmanni* and prey used in behavioral experiments: cockroaches (*Periplaneta americana*), crickets (*Acheta domesticus*) and spiders (*Ctenus* sp.).

Common name	Body part	Species	Mean length ± SD (mm)
Scorpion	Prosoma	*Tityus fuhrmanni*	5.39 ± 0.79
Crickets	Body length	*Acheta domesticus*	16.84 ± 1.5
Cockroaches	Body length	*Periplaneta americana*	16.68 ± 2.07
Spiders	Body length	*Ctenus* sp.	15.97 ± 2.38

### Prey acceptance and predatory behavior

For this experiment, the 33 collected individuals (9 males, 17 females and 7 juveniles), were used. Before starting the experiments, the level of hunger was standardized for all scorpions. First, individuals were fed with *Tenebrio molitor* (Linnaeus, 1758, Coleoptera: Tenebrionidae) larvae to satiety for one day. Subsequently, the individuals were deprived of food for 27 to 29 days. This period of starvation was chosen based on our preliminary observations with a different cohort of scorpions. 

Selected prey were randomly offered to scorpions following a random block design, where each prey individual is presented once to each scorpion individual, according to methods employed for similar predators such as spiders [41]. Random prey assignments were made using R software version 4.0.3 [42]. Before each experiment, both prey and scorpions were weighed and placed in an observation terrarium (21 cm x 15 cm x 6 cm height). Before the prey was introduced, scorpions remained in the observation terrarium for 20 minutes to allow them to habituate to the observation arena. Afterwards, prey was introduced at the opposite end of the scorpion’s location in the observation arena. All experiments were recorded using a Canon Powershot sx 160 IS camera under red light illumination. This was done to avoid scorpion disturbance, given that red light is not perceived by scorpions [43]. In each experiment, we recorded interactions between scorpions and their prey for 40 minutes. If during that time prey was captured, it was considered accepted, otherwise it was considered rejected. All experiments were made during night since we recorded a highest activity for *T. fuhrmanni* during these period based on preliminary observations. Prey acceptance was compared using a Generalized Estimating Equation [44], with binomial distribution, with the prey type, starvation time and scorpion group (males, females and juveniles) as explanatory variables and the prey consumption as response variable. Individuals were included as a random variable. Mean length of the offered prey is described in Table 1. 

We also recorded the number of attempts made by the scorpion before capturing the prey, defined as the number of times that scorpion tried to capture the prey using the pedipalps and their duration. Once captured, we also recorded the time it took for the scorpions to find a site to sting the offered prey, which started when the stinger first contacted prey’s body. We measured the number of stings, their duration and the immobilization time, which was considered as the time from the first sting until the prey stopped moving. When analyzing the immobilization time, we used: number and duration of stings, prey:predator mass ratio and prey type as explanatory variables. The data was analyzed using a Generalized Estimating Equation with Gamma distribution using the immobilization time as response variable and the remaining variables were used as explanatory variables. 

### Venom extraction

To evaluate the potency of the venom against different types of prey, we obtained venom from twenty-two scorpions of *T. fuhrmanni*. We selected the largest individuals for each group, namely nine males, nine females, and four juveniles out of 33 individuals used for behavioral experiments. Given the low number of individuals, venom was pooled. Before conducting the experiments, scorpions were kept under laboratory conditions and fed with larvae of *Tenebrio molitor.* Venom was obtained according to the methodology of González-Gómez et al. [30], using a 12V electro-stimulator with a square wave signal at 40Hz and a duty factor of 10%. Both electrodes were applied at the metasoma, so that no current passes through vital organs or through the stinger, to avoid damage to the individual, or altering venom properties. Once extracted, the venom was stored at a temperature of -20 °C and lyophilized [45]. This research was approved by Ethics Committee for Animal Experimentation (CEEA), University of Antioquia Rectoral Resolution 18084, No. 123-2019.

### Toxicity bioassays

To determine the median lethal concentration (LD_50_) for the evaluated prey against *T. fuhrmanni* venom*,* we made preliminary observations using the same prey offered in behavioral experiments (n = 8 individuals per each prey species). We used concentrations of 1.24; 2.5; 5; 10; 20; 30 and 40 μg/μL, based on reported LD_50_ values of different scorpion species used against arthropods [46, 47, 48]. 

In order to regulate the dosage of the venom, we used insects and spiders with similar masses (variation coefficient < 6%). Mean mass including variation coefficient and venom concentration are shown in Table 2. We selected consumed prey species instead of model organisms such as mice or *Drosophila* flies, since the latter may not be ecologically relevant for scorpions and produce biased results [49].

**Table 2.** Mean body mass and coefficient of variation for prey used in LD_50_ experiments. Used prey were: cockroaches (*Periplaneta americana*), crickets (*Acheta domesticus*) and spiders (*Ctenus* sp.), including the number of used individuals (n).

Common name	Species	Concentration (μg/μL)	Mean weight ± SD (g)	Coefficient of variation	n
Cockroach	*Periplaneta americana*	0	0.35 ± 0.017	5.39	28
		20	0.36 ± 0.020	5.71	15
		40	0.34 ± 0.020	5.93	15
		60	0.35 ± 0.016	4.75	15
		80	0.37 ± 0.018	4.98	10
Cricket	*Acheta domesticus*	0	0.35 ± 0.019	5.39	28
		10	0.36 ± 0.014	3.94	15
		20	0.38 ± 0.020	4.79	15
		30	0.40 ± 0.020	5.46	15
		40	0.37 ± 0.020	4.92	15
Spider	*Ctenus* sp.	0	0.35 ± 0.013	3.78	28
		10	0.36 ± 0.017	4.33	15
		20	0.36 ± 0.015	4.25	15
		30	0.35 ± 0.014	3.85	15

In each trial, we randomly selected one of the prey types, and the prey was exposed to a temperature of 0°C for approximately 30 seconds to put it in a temporary state of torpor. Then, a volume of 5 μL of venom solution, dissolved in physiological saline (0.9% NaCl in purified water), was injected [50, 51]. This procedure was repeated for all different concentrations and prey types (Table 2). As a control group we injected physiological saline solution without venom, we used 28 individuals in the control group for each prey type. 

Venom injection was made in a place where no vital parts of prey were affected. It was applied in the coxa joint of leg III of the insects, while in the spiders the application was made in the coxae of leg IV. Application placement was chosen based on previous evidence which suggests that the process of application in the coxae joints itself does not cause death [15]. The injections were made with a 10 μL Hamilton syringe. After the injection, individuals were placed in plastic boxes, with shelter and water *ad libitum*. Observations were made continuously for the first two hours to describe initial symptoms after injection, such as partial paralysis or tremors. At 24 hours and again at 48 hours after injection we recorded the number of dead individuals.

For the analysis of the LD_50_, we used a binomial generalized linear model with the individual’s survival as response variable and prey type, and log-transformed concentration as explanatory variables with probit function as link [52]. 

## Results

### Prey acceptance and predatory behavior

**Figure 1.**
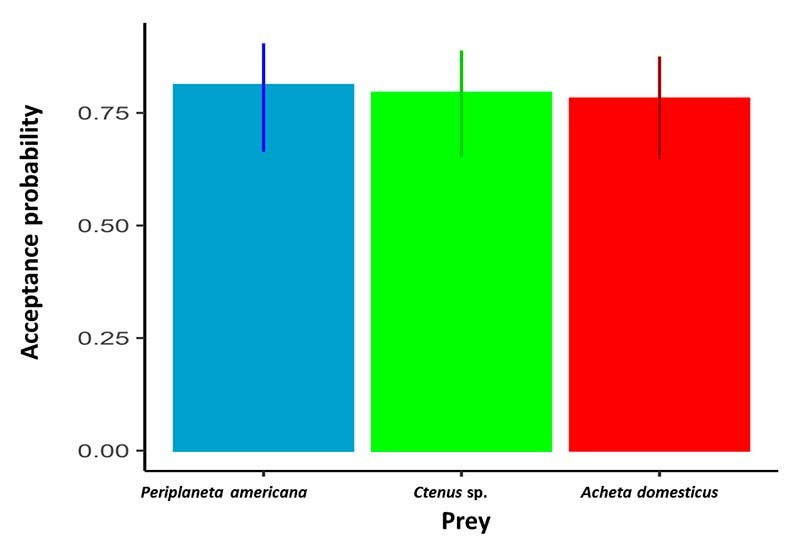
Acceptance probability of *Tityus fuhrmanni* of spiders (*Ctenus* sp.), crickets (*Acheta domesticus*) and cockroaches (*Periplaneta americana*). Bars represent means, lines are 95% confidence intervals. Mean values and confidence intervals were estimated using a generalized estimating equation with a binomial distribution.

**Figure 2.**
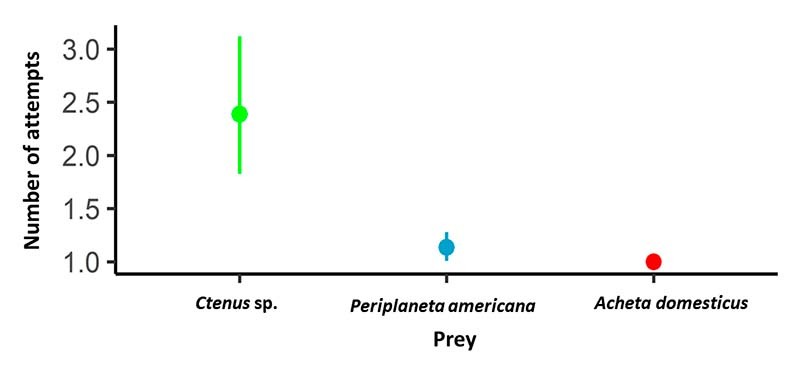
Number of attempts employed by *Tityus fuhrmanni* when capturing spiders (*Ctenus* sp.), crickets (*Acheta domesticus*) and cockroaches (*Periplaneta americana*). Points represent means, lines are 95% confidence intervals. Mean values and confidence intervals were estimated using a generalized estimating equation with a Poisson distribution.

We did not find a preference in *T. fuhrmanni* for any of the prey (χ^2^ = 0.92, df = 1, p = 0.62), or between males, females and juveniles (χ^2^ = 2.35, df = 2, p = 0.12). We also did not find a significant effect of starvation time on acceptance (χ^2^ = 2.72, df = 1, p = 0.09). Acceptance for all offered prey was higher than 70% (Figure 1). However, the number of capture attempts was significantly higher (χ^2^ = 48.50, df = 2, p < 0.01) for spiders compared to other prey such as cockroaches (contrasts: p < 0.01) and crickets (contrasts: p < 0.01). In the majority of cases (74%), scorpions pinched spiders by their legs, which were autotomized allowing them to escape (Additional file 1). In addition, the spiders made rapid escape movements and sometimes tried to bite the scorpions when attacked. Significant differences were also found between cockroaches and crickets (contrasts: p = 0.04). The number of capture attempts was higher for cockroaches (Figure 2), which made fast evasive movements when attacked, while crickets were quickly subdued (see Additional files 2 and 3).

**Figure 3.**
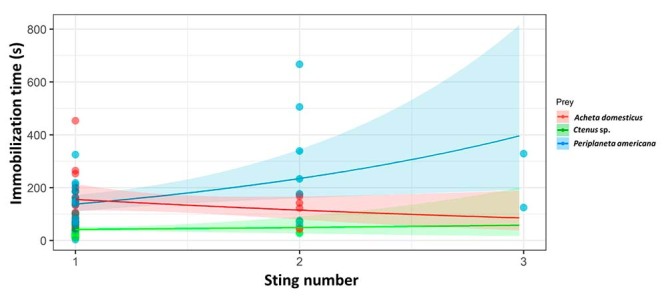
Relationship between immobilization time and sting number of *Tityus fuhrmanni* when capturing spiders (*Ctenus* sp.), crickets (*Acheta domesticus*) and cockroaches (*Periplaneta americana*). Shaded bands represent confidence intervals. Lines and confidence intervals were estimated using a generalized estimating equation with a gamma distribution.

When evaluating the immobilization time, we found that neither the body mass of the prey (*X* ^*2*^ = 0.6, df = 1, p = 0.42), nor the total duration of stinging (χ^2^ = 0.4, df = 1, p = 0.50) had a significant effect on the immobilization time. The immobilization times were significantly different between prey (χ^2^ = 83.7, df = 2, p < 0.01), being lower in spiders compared to crickets and cockroaches. No significant differences were observed between the remaining prey (Figure 3). We also found significant differences in immobilization times regarding the number of stings (χ^2^ = 9.6, df = 1, p = 0.02). It is noteworthy that spiders and crickets received two stings at most to be paralyzed, while some cockroaches needed up to three stings before being immobilized. We found a significant effect of the interaction between the type of prey and the number of stings on the immobilization time (χ^2^= 7.0, df = 2, p = 0.03). The interaction occurred because the immobilization time decreased with the number of stings in crickets, while it increased in cockroaches and showed a slight increase in spiders.

### Toxicity bioassays

**Figure 4.**
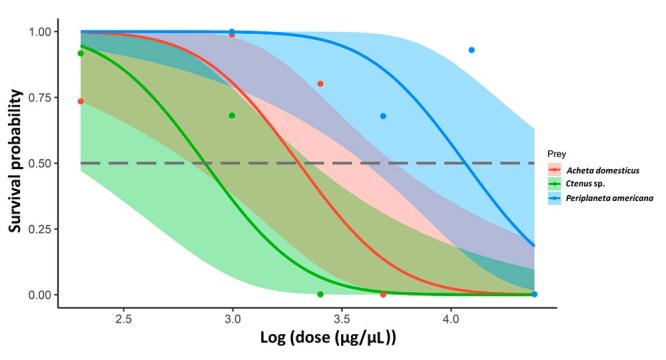
The survival curves as a function of log(dose). Median lethal dose expressed as mg of venom per kg of prey are expressed in Table 3. Shaded bands represent confidence intervals. All parameters were estimated using a binomial generalized linear model.

Toxicity was significantly different between prey types (χ^2^ = 60.60, df = 2, p = 0.038), we also recorded a significant effect of dosages on mortality (χ^2^ = 36.50, df = 1, p < 0.039). When evaluating differences in prey type, we found that cockroaches were the most resistant prey to *T. fuhrmanni* venom, followed by crickets, with values close to significance (p = 0.07) but more resistant than spiders (contrasts: p = 0.027). We found that spiders and crickets were similarly affected by the scorpion venom (contrasts: p = 0.32). None of the individuals in the control group died for any of the prey types. The LD_50_ curves are reported in Figure 4, while LD_50_ values are shown in Table 3. Although not quantified, behavioral effects of venoms were observed shortly after injection, including tremors in all prey, as well as vomiting in crickets.

**Table 3.** Estimated LD_50_ values for each prey type.

Common name	Species	LD_50_ (Log(dose) ± standard error)	LD_50_ (mg/kg)
Spider	*Ctenus* sp.	2.88 ± 0.02	249.91
Cricket	*Acheta domesticus*	3.30 ± 0.02	376.35
Cockroach	*Periplaneta americana*	4.09 ± 0.02	846.07

## Discussion

Our results indicate that the scorpion *T. fuhrmanni* can overcome and feed on prey types with contrasting morphologies such as cockroaches, crickets and spiders. The fact that the scorpions consumed all offered prey types in similar proportions suggests they do not have a specific preference. A similar trend was observed for males, females and juveniles, which accepted prey in very similar proportions. However, given the limited number of individuals used, further studies should explore if prey acceptance varies according to the instar on this species. The ability to capture prey with contrasting morphologies observed in *T. fuhrmanni* agrees with previous studies, which describe other scorpion species as being dietary generalists [23, 27, 28, 29] However, further studies evaluating capture of prey with different morphologies to confirm the trends observed in our study, may be valuable. All the accepted prey have been previously reported as common prey for other scorpion species under natural conditions. Given their sympatric habits, these prey types are likely also an important part of the diet of *T. fuhrmanni*. 

Interestingly, we found that *T. fuhrmanni* employs a higher number of prey capture attempts when preying on spiders. This agrees with the report by Simone et al [23], who found that bothriurid scorpions needed more attempts to capture spiders compared to other prey. Probably the higher number of attempts recorded in spiders may be due to appendage autotomy, which is a defensive strategy frequently used by spiders [53]. Wolf spiders for instance, increase their survival probabilities by autotomizing some legs when attacked by scorpions [54]. Our results corroborate that autotomy may be an effective defensive strategy for spiders when attacked by scorpions, since the ctenid spiders used in our study often removed their legs when attacked by scorpions, and these proceeded to consume them before a next attempt at capture. In addition, some of the spiders tried to bite the scorpions while being attacked and displayed fast bouts of locomotion when the scorpion approached. Nevertheless, we did not record any successful bite of the spiders towards the scorpions. These behaviors combined might explain the high number of attempts necessary for scorpions to capture spider prey. Interestingly, cockroaches were the second hardest prey to capture for the scorpions, which might be explained by their fast movements. The high speed of cockroaches makes them a difficult prey for some predators like frogs [55]. 

We expected that longer stinging durations would allow the scorpions to inject more venom, which would be reflected in shorter immobilization times. However, this was not the case. We suppose that the lack of relationship between immobilization time and duration of sting could have occurred since some of the stings might have not been effective or may even have been “dry”, as reported in some species which use defensive stings [21,23], or to minimize venom expenditure such as has been shown for some scorpion species [17]. In contrast, we found a significant interaction between the number of stings and the immobilization times and prey type. During behavioral experiments, both spiders and crickets needed up to two stings to be paralyzed. In the case of crickets, the immobilization time decreased as the number of stings increased. However, although showing low immobilization times, we found a slight correlation between the immobilization and the number of stings in *Ctenus* sp. This is probably a consequence of the spider defensive behavior that prevented an efficient injection of venom in some cases. During the venom injection bioassays, we found that both spiders and crickets are equally susceptible to scorpion venom and therefore this similar sensitivity to the venom may lead them to be paralyzed in similar times. It must be noted that LD_50_ does not directly record time to paralysis, but is used here as a general indication of sensitivity to the venom. Further studies regarding venom sensitivity should include prey paralysis time when different dosages are used. The immobilization times for the spiders during the behavioral experiments were shorter than for the other prey. We presume that this occurred because spiders are potentially dangerous prey, with a high retaliation capacity and therefore, they must be paralyzed quickly [56]. Potentially, scorpions used more venom against this prey, and/or the lower LD_50_ values for spiders resulted in faster immobilization after being stung. This has also been suggested for other venomous predators when subduing difficult prey. A reduced contact time between a predator and a potentially dangerous prey reduces the probability of injury to the former [57]. Interestingly, in cockroaches the number of stings increased with time of immobilization. These results are consistent with similar studies that show longer immobilization times for cockroaches when preyed upon by *Phoneutria* spiders [15]. We observed a similar trend, which we presume is due to the scorpion having difficulties when trying to sting the cockroaches, since the tough cuticle of this prey prevented the stinger from penetrating. A similar trend has been observed in some *Loxosceles* spiders, as well as in other scorpions, when attacking armored arthropods such as harvestmen [58]. Although we expected a significant effect of mass on immobilization time in scorpions, as in other predatory venomous arthropods such as spiders [15], this was not the case, probably as we used prey with similar mass ranges.

The LD_50_ results agreed with our immobilization time observations, since dosages needed to kill cockroaches after experimental injection were higher compared to spiders and crickets. Although venom resistance in cockroaches was similar to crickets, values close to significance suggest that by adding more individuals, significant differences might be found. A high resistance to venoms as well as other toxins has been reported for cockroaches [59, 60], including other scorpion species [61], which might also explain why scorpions needed more stings to subdue this prey type. In addition to the armored body of cockroaches, the venom resistance might explain why the number of stings was positively correlated with the immobilization time. In the case of crickets and spiders, toxicity assays showed similar values, but immobilization time was different for these prey when captured by the scorpion. These results indicate that scorpions are probably able to dose venom depending on the prey type. Given that spiders are a more dangerous prey than crickets, it is possible that scorpions inject more venom to paralyze this prey more quickly to prevent possible injuries. Such venom metering for more dangerous prey also occurs in other venomous predators such as snakes or spiders [10, 62]. Trends recorded in our study are similar to those recorded in other buthid species such as *Centruroides edwardsii* [61], where *Periplaneta* were also more resistant to scorpion venom than *A. domesticus* Interestingly LD_50_ values found in our study for *T. fuhrmanni* were lower for *A. domesticus* than other scorpions such as *C. edwardsii*. Although LD_50_ values might have been affected by using *T. fuhrmanni* specimens kept under laboratory conditions for long periods, our results are consistent with those reported by Gómez et al [33], who suggested that *T. fuhrmanni* venom is less toxic to insects than other buthid species. 

Given that the venom was able to incapacitate unrelated arthropods, such as insects and spiders, our results suggest it is not specific against a particular prey type group. However, these results should be interpreted carefully as differences in toxicity between crickets and spiders were close to significance. Additional studies should explore if this is the case for other buthid scorpions. Further studies should evaluate if the toxicity against vertebrates found in this scorpion genus might be due to selection for vertebrate prey, as has been suggested for other predatory arthropods, such as belostomatid bugs, spiders, and centipedes [13,15, 63], or even other scorpion species [61]. 

## Conclusion

Our results show that the scorpion *T. fuhrmanni* is a generalist and potentially euryphagous predator able to overcome and consume a variety of prey besides spiders. Although venom affected all evaluated prey, it had a different potency depending on the animal. It is probable that the scorpion is able to dose venom for potentially dangerous prey, which would be interesting to evaluate in future studies.

Traditionally, LD_50_ studies on venomous arthropods such as spiders or scorpions are focused on model organisms, like mice or *Drosophila* flies, which have little or no ecological relevance for the studied venomous animal [15, 50, 65]. In the present study we showed a link between toxicity and prey capture, using potentially sympatric prey of the scorpion *T. fuhrmanni*. Although one of the prey species we used was not sympatric - and local prey may have specific adaptations, such as resistance to venom that may not be evolved in laboratory-reared prey as occurs with some snake prey [65] - the other prey types were confirmed sympatric species. Evaluating toxicity using sympatric prey is particularly important. Toxicological arms-races between predator and local prey may explain the toxicity in medically important arthropods, as has been suggested for *Phoneutria* and *Latrodectus* spiders [15, 64]. These results show the importance of multidisciplinary studies that include both behavioral, as well as toxicological approaches, to understand predator-prey relationships. Further studies should also explore if a similar trend occurs in other scorpion species. Similarly, other aspects of the prey, such as mobility and metabolism should be considered in further toxicological studies, as these aspects may explain part of the effect of venom on different prey.

## Supplementary material

The following online material is available for this article:

Additional file 1. Video of the scorpion *Tityus fuhrmanni* capturing a *Ctenus* sp. spider.

Additional file 2. Video of the scorpion *Tityus fuhrmanni* capturing an *Acheta domesticus* cricket.

Additional file 3. Video of the scorpion *Tityus fuhrmanni* capturing a *Periplaneta americana* cockroach.
