# Supplementary material for: Linking toxicity and predation in a venomous arthropod: the case of Tityus fuhrmanni (Scorpiones: Buthidae), a generalist predator scorpion
Source: J Venom Anim Toxins Incl Trop Dis. 2022 Jan 7;28:e20210036. doi: 10.1590/1678-9199-JVATITD-2021-0036 (PMC8747031; doi:10.1590/1678-9199-JVATITD-2021-0036)
Supplement: Additional file 2. [file 1678-9199-jvatitd-28-e20210036-s2.zip › 1678-9199-jvatitd-28-e20210036-s2.pdf]

J Venom Anim Toxins incl Trop Dis

https://doi.org/10.1590/1678-9199-JVATITD-2021-0036

Supplementary Material to "Linking toxicity and predation in a venomous arthropod: the case of *Tityus fuhrmanni* (Scorpiones: Buthidae), a generalist predator scorpion"

Additional file 2. Video of the scorpion *Tityus fuhrmanni* capturing an *Acheta domesticus* cricket.
